# The Book World of Medicine and Science

**Published:** 1906-01-06

**Authors:** 


### THE BOOK WORLD OF MEDICINE AND SCIENCE.

Therapeutics: Its Principles and Practice. By Horatio C. Wood and H. C. Wood, Jun., of the University of Pennsylvania. Twelfth Edition. (Philadelphia and London: J. B. Lippincott Company, 1905. Pp. 907. Price 18s. net.)

There are many changes in and additions to the twelfth edition of this treatise on therapeutics. The recent publication of the eighth edition of the United States Pharmacopœia is to a large extent the cause. Over seventy new drugs are discussed, and the chapters on disinfectants and expectorants have been re-written, rearranged, and reclassified. The work is an exhaustive one; it deals thoroughly with the physiological and therapeutic action of drugs. Remedies and remedial measures, such as hydrotherapy and electricity, which are not drugs, occupy a very small part of the book. The volume is of most value as a text book and work of reference to the American student and practitioner; among medical men in this country it will be especially useful to those engaged in pharmacological research.

Psychological Medicine. By Maurice Craig, M.A., M.D., M.R.C.P. (London: J. and A. Churchill. 1905. Pp. 450. Twenty-two illustrations. Price 12s. 6d.)

In his second chapter Dr. Craig tells us, truly enough, that insanity, like sanity, is indefinable; and in this connection he gives the medical witness a caution not to commit himself to an answer in the terms of the question should counsel ask for a definition of the words. At the same time we are presented with a really good definition, that is a definition which supplies us with all we really require either for giving evidence in a court of justice or for comprehending the thesis of his work. The chapter on the causation of insanity is not a long one; but it is clearly expressed, and its succinctness does not prevent its covering in its ten pages all the well-known, well-trodden ground. Dr. Craig does not look upon religion as a very common cause of insanity, remarking that in the vast majority of cases religion, *per se*, does not "produce" insanity. We are disposed to agree with him, although much would depend upon the exact meaning of the word "produce"; and we are not told by Dr. Craig how far the ordinary intellect may be dwarfed by the unquestioning faith demanded by some religious bodies, and how far this cramping may prevent the patient shaking off what might be a slight illness, but which under the circumstances develops into a serious one. Perhaps that is a subject for some future inquirer. One of the best sections is that on general paralysis. It is most comprehensive; and as we read it we felt convinced that an accurate knowledge of what is therein written would often free the general practitioner from diagnostic errors when his patient is in an early stage of the disease, and when so much may depend upon the recognition and significance of this disease. We regret that the space at our disposal for this notice forbids our doing more than naming and commending such chapters as are headed "Intoxication Psychoses," "Feigned Insanity," "Sleeplessness," "Insanity and Physical Disease," "Relationship of Insanity with Law," and many others. Taking it as a whole there is an eminently practical tone resonant throughout the volume. Opinions of other men are carefully weighed and fairly stated; and while the author does not sink his own views he is never dogmatic, hence this work of Dr. Craig's may be confidently recommended both to the student and to the practitioner. The former will find it ample for any examination, and the latter may think that its 400 pages of clear type are about as much as he can be expected to digest amid the plethora of volumes on all possible subjects presented to him nowadays.

